# Case Report: COVID-19 Recovery from Triple Infection with *Mycobacterium tuberculosis*, HIV, and SARS-CoV-2

**DOI:** 10.4269/ajtmh.20-0756

**Published:** 2020-08-18

**Authors:** Neyla Rivas, Mario Espinoza, Alejandra Loban, Odemaris Luque, Julio Jurado, Nicolás Henry-Hurtado, Amador Goodridge

**Affiliations:** 1 Servicio de Medicina Interna, Complejo Hospitalario Dr. Manuel Amador Guerrero, Caja de Seguro Social, Colón, Panama;; 2 Programa Regional de Control de Tuberculosis, Ministerio de Salud, Colón, Panama;; 3 Tuberculosis Biomarker Research Unit, Instituto de Investigaciones Científicas y Servicios de Alta Tecnología-INDICASAT-AIP, City of Knowledge, Panama City, Panama

## Abstract

COVID-19, designated as SARS-CoV-2, has caused millions of infections worldwide, including in patients with concomitant infections. Here, we report two unusual cases of patients with triple infections of SARS-CoV-2, *Mycobacterium tuberculosis*, and HIV. Both cases were confirmed through microbiological and immunological studies. The acute respiratory phase in both patients was treated with supplemental oxygen. Antituberculosis and antiretroviral therapies were started simultaneously. In 2 weeks, both patients demonstrated clinical improvement and recovery from COVID-19. Our findings suggest that even in cases of triple infection, clinical management together with respiratory therapy contributes to patient survival.

## CASE REPORT

A 29-year-old man from the coastal area of the province of Colón, Panama, arrived at the end of March with a 2-week history of a nonproductive cough. In addition, he had had moderate exertional dyspnea, asthenia, adynamia, and weight loss of about 30 pounds in the previous 5 months. There was no history of fever. Being a patient with an acute respiratory condition and suspicion of COVID-19, we analyzed a nasopharyngeal swab by real time PCR (RT-PCR) and confirmed a SARS-CoV-2 infection.

**Figure 1.**
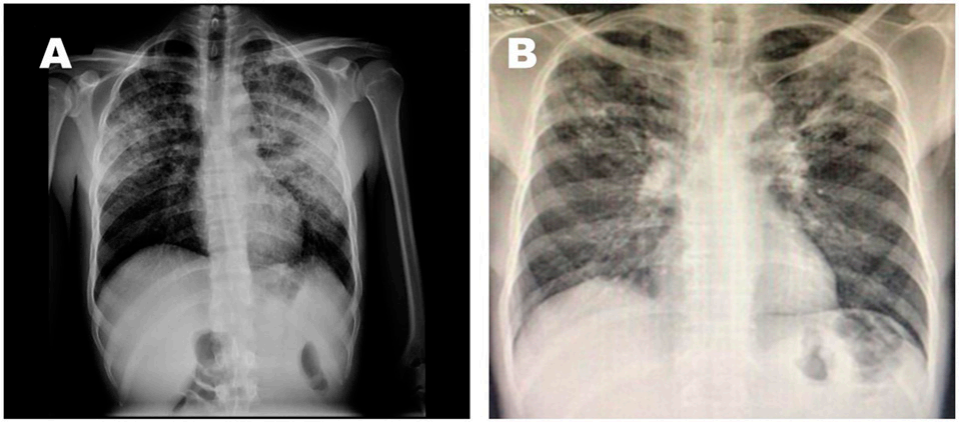
Thorax radiography: Patient (**A**) demonstrates bilateral interstitial infiltration. Patient (**B**) demonstrates left apical interstitial infiltration.

He was admitted to the reference hospital for symptoms suggestive of pneumonia secondary to COVID-19 and tuberculosis with a confusion, urea, respiratory rate, blood pressure, and 65 years of age or older score of 1 and a National Early Warning Score 2 of seven points. At the time of hospital admission, the patient showed an absence of leukocytosis, with mild neutrophilia (80%) and marked lymphopenia (4.1%), and mild anemia (9.9 g/dL) without alterations in the platelet series. C-reactive protein was increased (184.7 mg/L), ferritin was elevated (> 2,000 ng/dL), D-dimer reached 4,600 ng/mL, and procalcitonin was 5.76 ng/mL; unfortunately, interleukin six testing was not available in our hospital. The patient’s respiratory frequency was 35, and his oxygen saturation was less than 90%. Consequently, he was transferred to the intensive care unit where he received invasive mechanical ventilatory support. See Figure 1A.

On admission, an outpatient sputum analysis by GeneXpert MTB/RIF assay resulted positive for infection with *Mycobacterium tuberculosis* without resistance to rifampicin. We also noted an associated empyema. In addition, routine evaluation with confirmatory tests showed coinfection with HIV, with a viral load of 78,100 copies/mL and a CD3/4^+^ level of 133 C/µL. We initiated first-line antituberculosis therapy, including isoniazid, rifampin, ethambutol, and pyrazinamide. We supplemented with intravenous levofloxacin in a dose of 750 mg every day for 14 days and 500 mg of azithromycin given orally every day for 5 days. In addition, we administered hydroxychloroquine (HCQ) orally in doses of 400 mg every 12 hours for the first day and then 200 mg every 12 hours for 10 days. Low molecular weight heparin at a dose of 1 mg/kg was administered subcutaneously every day as a prophylaxis for prothrombotic events. Ten days later, the patient was stable after extubation. He was then transferred from the COVID ward to a tuberculosis isolation room. The patient’s temperature remained greater than 37°C, and he presented overall weakness. A chest computed tomography revealed a pleural empyema. Later on, he developed nosocomial pneumonia. We started antiretroviral therapy (ART) with tenofovir, lamivudine, and dolutegravir; no adverse effects were reported. On day 14 after admission, we evaluated two nasopharyngeal swabs by RT-PCR and did not detect SARS-CoV-2. A second test administered 48 hours later failed to detect the virus, confirming recovery from COVID-19 according to the laboratory criteria. The patient was discharged from the hospital 2 months later after recovery from nosocomial pneumonia.

The second case refers to a 53-year-old man from the city of Colón who arrived at the end of April with a history of pulmonary tuberculosis and in his second phase of treatment with rifampicin and isoniazid. This patient reported approximately 7 days of unquantified fever, with no predominance of hours, in addition to dyspnea on moderate exertion, asthenia, and adynamia. On arrival at the emergency department, he was hemodynamically stable, but tachypneic, with respiratory rates ranging from 28 to 30 breaths/minute; in addition, he presented bilateral crepitus of apical predominance. These findings supported hospital admission as a suspected COVID-19 case. A nasopharyngeal swab analyzed by RT-PCR confirmed a SARS-CoV-2 infection. The patient was transferred to a respiratory isolation room to receive management for COVID-19 pneumonia, with oxygen at 4 L/min via nasal cannula, and continue his treatment for pulmonary tuberculosis.

On admission, we confirmed coinfection with HIV with a CD4^+^ level of 294 C/µL and a viral load of 461,000 copies/mL. The hemogram did not indicate leukocytosis or alterations in the platelet count; however, the patient did show mild anemia (hemoglobin 10.8 g/dL). There were no alterations in the patient’s kidney function or liver function tests. An arterial blood gas test showed a pH of 7.43, PCO_2_: 32, PO_2_: 69, HCO_3_: 24, lactate: 1, and oxygen saturation of 94%. A chest radiograph showed a left apical infiltrate. D-dimer and IL-6 testing were not available (Figure 1B).

During hospitalization, the patient was initially managed with oxygen therapy with a nasal cannula at 4 L/min, obtaining saturations of 98% and decreased dyspnea. In addition, he received treatment with broad-spectrum antibiotics as follows: 2 gr IV ceftriaxone per day for 7 days and 500 mg azithromycin given orally once a day for 3 days. Thromboprophylaxis was started with 40 mg enoxaparin given subcutaneously every day. In addition to the aforementioned, the patient received HCQ in a dose of 400 mg twice a day for 1 day and then 200 mg every 12 hours for 5 days. On the fourth day, the patient was stable and did not require oxygen therapy. The patient showed improvement in his general condition, and on the seventh day of his hospitalization, which was more than 14 days from the onset of symptoms, he was considered clinically recovered and discharged with follow-up on an outpatient basis.

## DISCUSSION

Here, we describe two cases of an unusual triple infection with *M. tuberculosis*, HIV, and SARS-CoV-2 in Panama. We describe the clinical management, therapy, and progression of both cases that allowed for recovery from COVID-19. On admission, the standard of care for COVID-19 patients in this hospital included critical status assessment, invasive mechanical ventilation for severe cases, and clinical management with oxygen by the nasal canula for mild and moderate cases. In these two reported cases, acute respiratory failure triggered by COVID-19 was promptly addressed. This strategy facilitated rapid recovery despite a triple infection. We used HCQ to treat both cases as recommended by the WHO Solidarity clinical trial, which was ongoing at the time of the patients’ admission. Unfortunately, recent evidence has raised concerns about HCQ, and the WHO has recommended against its use.^1^ To date, there have been no similar reports of patients presenting with three concomitant infectious diseases. However, reports describe cases of people living with HIV affected by previous SARS, Middle East respiratory coronavirus, and COVID outbreaks. These case reports of people coinfected with HIV affirm that these patients recover most of the time. Although other authors have separately reported coinfection with tuberulosis (TB),^2^ our study analyzed the progression of triple infection during the COVID pandemic.

Coinfection with HIV and SARS-CoV-2 does not appear to worsen COVID-19 infection outcomes. The greatest number of complicated COVID-19 cases occurs in patients with chronic disease comorbidities, such as hypertension, diabetes, and cancer, in addition to those older than 60 years. However, the evidence is not sufficient to indicate whether mortality in patients with a severe immunosuppressed state is higher or lower.^3^ For example, a study by Guo et al.^4^ in China concluded that the incidence of COVID-19 in people living with HIV is comparable to that of the general population. In addition, this study indicated that low CD4^+^ levels and high levels of viral load influence the lethal progression of COVID-19. For their part, Harter et al.^3^ in Germany documented that of 32 patients with double infections of HIV and SARS-CoV-2, only 9% died and 76% of these cases were reported as mild. Despite these findings, Vizcarra et al.^5^ in Spain suggest that people living with HIV should not consider themselves protected from SARS infection-CoV-2 or having a lower risk of serious disease. In fact, there are not yet enough data to test or rule out this hypothesis.^6^ More recently, Gervasoni et al.,^7^ in Italy, found a 96% survival of HIV patients with SARS-CoV-2 coinfection. They argued that ART may have played a role in the positive evolution of COVID-19 in their patient study group. In fact, Del Amo et al.^8^ in Spain demonstrated that ART based on nucleotide reverse transcriptase inhibitors may protect patients from acquiring COVID-19. They noted that those HIV patients receiving tenofovir disoproxil fumarate (TDF)/emtricitabine (FTC) had a lower risk for COVID-19 and related hospitalizations than those receiving other ART drugs.

The SARS-CoV-2 and *M. tuberculosis* coinfection situation is poorly understood. An initial preliminary report from Liaoning Province, China, indicated that 36% of COVID-19 cases had a current TB infection or had been infected in the past. This was significantly higher than the percentage of other comorbidities, including diabetes, hypertension, coronary heart disease, and chronic obstructive pulmonary disease (25%, 22.2%, 8.33%, and 5.56%, respectively).^9^ By contrast, a study on COVID-19 and coinfection rates with other respiratory pathogens did not include an analysis of the presence of *M. tuberculosis* as a causal agent.^10^ However, Tadolini et al.^2^ did report the clinical characteristics and progression of a series of 49 cases of TB and COVID-19 coinfection in eight Global Tuberculosis Network countries, including Belgium, Brazil, France, Italy, Russia, Singapore, Spain, and Switzerland. In this study, one-third of the SARS-CoV-2 coinfections were detected during TB treatment. Certainly, the detection of COVID-19 facilitated the diagnosis of TB. Unfortunately, Tadolini et al.^2^ failed to determine the association between SARS-CoV-2 infection and progression from latent tuberculosis infection to active disease. Only recently, an expert panel published a rapid consensus document on the epidemiology and immunology of viral infections and their interactions with TB.^11^ Together, these reports support the need for further larger studies to understand the physiopathology during *M*. *tuberculosis*, and SARS-CoV-2 coinfection. Triple infection with SARS-CoV-2, *M. tuberculosis*, and HIV is unusual. We believe our study is the first report of patients with a triple infection during the COVID-19 pandemic. In fact, Latin America accounted for 11.2% of HIV-positive TB patients, or approximately 4.6 per 100,000 people, for the year 2017.^12^ Unfortunately, it is estimated that the mortality rate of people coinfected with TB and HIV has remained at 20% since 2012 in the Latin American region.^13^ The mortality from triple infection in patients with COVID-19 has not been reported. Recently, a preliminary report from the South African province of Western Cape analyzed the results of 12,987 patients with COVID-19.^14^ After adjusting for other risk factors, they found that HIV increased the risk of death in COVID-19 patients by a factor of 2.75, whereas active TB increased the risk by a factor of 2.58. This study did not provide data for patients with both HIV and TB. The cases we reported in our study survived COVID-19. Population-based studies will be key to accurately measuring the risk of death for triple infection.
